# Graded Intensity Aerobic Exercise to Improve Cerebrovascular Function and Performance in Older Veterans: Protocol for a Randomized Controlled Trial

**DOI:** 10.2196/58316

**Published:** 2024-09-26

**Authors:** Medina Oneyi Bello, Kevin Michael Mammino, Mark Anthony Vernon, Daniel G Wakeman, Chanse Aerius Denmon, Lisa Crystal Krishnamurthy, Venkatagiri Krishnamurthy, Keith Matthew McGregor, Thomas Samuel Novak, Joe Robert Nocera

**Affiliations:** 1 Joseph Maxwell Cleland Atlanta Veteran Affairs Medical Center Decatur, GA United States; 2 School of Medicine Emory University Decatur, GA United States; 3 Department of Physics and Astronomy Georgia State University Atlanta, GA United States; 4 Birmingham Veteran Affairs Medical Center Birmingham, AL United States

**Keywords:** aerobic exercise, exercise, functional magnetic resonance imaging, fMRI, veterans, quality of life, sedentary lifestyle, elderly, geriatrics, geriatric, older adults, cardiovascular disease, health promotion, aging, cognitive, cognitive health, physical health

## Abstract

**Background:**

Growing health care challenges resulting from a rapidly expanding aging population necessitate examining effective rehabilitation techniques that mitigate age-related comorbidity and improve quality of life. To date, exercise is one of a few proven interventions known to attenuate age-related declines in cognitive and sensorimotor functions critical to sustained independence.

**Objective:**

This work aims to implement a multimodal imaging approach to better understand the mechanistic underpinnings of the beneficial exercise-induced adaptations to sedentary older adults’ brains and behaviors. Due to the complex cerebral and vascular dynamics that encompass neuroplastic change with aging and exercise, we propose an imaging protocol that will model exercise-induced changes to cerebral perfusion, cerebral vascular reactivity (CVR), and cognitive and sensorimotor task-dependent functional magnetic resonance imaging (fMRI) after prescribed exercise.

**Methods:**

Sedentary older adults (aged 65-80 years) were randomly assigned to either a 12-week aerobic-based interval-based cycling intervention or a 12-week balance and stretching intervention. Assessments of cardiovascular fitness used the YMCA submaximal VO_2_ test, basal cerebral perfusion using arterial spin labeling (ASL), CVR using hypercapnic fMRI, and cortical activation using fMRI during verbal fluency and motor tapping tasks. A battery of cognitive-executive and motor function tasks outside the scanning environment will be performed before and after the interventions.

**Results:**

Our studies and others show that improved cardiovascular fitness in older adults results in improved outcomes related to physical and cognitive health as well as quality of life. A consistent but unexplained finding in many of these studies is a change in cortical activation patterns during task-based fMRI, which corresponds with improved task performance (cognitive-executive and motor). We hypothesize that the 12-week aerobic exercise intervention will increase basal perfusion and improve CVR through a greater magnitude of reactivity in brain areas susceptible to neural and vascular decline (inferior frontal and motor cortices) in previously sedentary older adults. To differentiate between neural and vascular adaptations in these regions, we will map changes in basal perfusion and CVR over the inferior frontal and the motor cortices—regions we have previously shown to be beneficially altered during fMRI BOLD (blood oxygen level dependent), such as verbal fluency and motor tapping, through improved cardiovascular fitness.

**Conclusions:**

Exercise is one of the most impactful interventions for improving physical and cognitive health in aging. This study aims to better understand the mechanistic underpinnings of improved health and function of the cerebrovascular system. If our hypothesis of improved perfusion and cerebrovascular reactivity following a 12-week aerobic exercise intervention is supported, it would add critically important insights into the potential of exercise to improve brain health in aging and could inform exercise prescription for older adults at risk for neurodegenerative disease brought on by cerebrovascular dysfunction.

**Trial Registration:**

ClinicalTrials.gov NCT05932069; https://clinicaltrials.gov/study/NCT05932069

**International Registered Report Identifier (IRRID):**

DERR1-10.2196/58316

## Introduction

The rapid growth of the aging global population introduces significant health care and fiscal challenges due to an increased prevalence of age-related neurodegenerative disease and disability. For example, of the 60 million Americans over 65 years of age, it is estimated that 1 in 3 will be diagnosed with Alzheimer disease (AD) or a related dementia in their lifetime. Intertwined with aging-related pathologies are physiological changes in cerebrovascular function [1]. In fact, recent epidemiological and clinicopathological data indicate considerable overlap between cerebrovascular dysfunction in aging and AD [2,3]. While AD is an overt pathology, its “subclinical” progression appears to take decades before crossing a diagnostic threshold [4]. This subclinical stage spans a significant time frame for targeted interventions aimed at limiting vascular dysfunction in high-risk older adults.

It has been demonstrated that of all modifiable risk factors for dementing illness, decreasing sedentary behavior is the most statistically significant and effective measure to counter disease progression and associated cognitive decline in older adults [5]. For example, nearly a quarter million cases of AD could be prevented in the United States alone by improving the cardiovascular fitness profile in older adults. This staggering number is an exciting target not only for prevention during aging but also to better understand aging processes and their underlying mechanisms that are malleable to exercise. Equally significant, this number probably underestimates the total impact of increasing cardiovascular fitness due to its effects on other risk factors in aging, including hypertension and obesity [6].

This study will explore the impact of aerobic exercise on cerebrovascular health in older adults. Research over the last few decades has driven the continual promotion of exercise as one of the most impactful interventions for central nervous system health and function [7-9]. We know that older adults who are physically active have improved peripheral vascular health, but the impact of an exercise intervention on cerebrovascular health is less known. We will fill this gap by examining changes in basal cerebral perfusion and cerebral vascular reactivity (CVR) in older adults following a proven cardiovascular fitness intervention [10-12]. If our hypotheses of improved perfusion and CVR are supported, it would inform intervention strategies and add important new information about the potential of exercise to improve brain health in aging. This would have immediate implications for older adults at risk for neurodegenerative disease brought on by cerebrovascular dysfunction.

## Methods

### Overview

This study design followed a parallel group design where participants engaged in exercise for 12 weeks.

### Participants

Older adult participants were pseudorandomized to 1 of two 12-week interventions: interval-based aerobic “spin” cycling exercise or nonaerobic balance and stretching exercise to serve as an active control condition. Each intervention was group-based and in-person, with trained personnel leading each class. Before beginning any portion of this study, we had to receive written approval from a qualified physician for participation in the fitness assessment and exercise intervention.

The key inclusion criteria and final participant pool will consist of right-handed, English-speaking individuals aged 65 to 80 years. Participants must also self-report a sedentary lifestyle defined as not participating in at least 30 minutes of moderate-intensity physical activity on at least 3 days/week for at least 3 months. Additionally, participants will not have dementia or any deficits in cognitive-executive function, as indicated by a Montreal Cognitive Assessment score (MoCA) score ≥26. Those with severe diabetes requiring insulin will also be excluded. However, individuals with controlled diabetes who meet our inclusion criteria for sedentariness and cognitive function will be allowed to participate. Individuals with any conditions contraindicated by magnetic resonance imaging (MRI) acquisition (including but not limited to ferrous metal implants, cardiac pacemakers or similar devices, claustrophobia, and morbid obesity) and those with any history of major psychiatric disorder (including but not limited to psychosis, major depression, and bipolar disorder) will also be excluded.

### Measures

#### Overview

Pre- and posttests of cardiovascular fitness, cognitive and motor function, and brain imaging will be conducted over 2 days (<3 hours per day) within a 2-week time frame.

#### Day 1: Cognitive/Motor and Physical Function Assessments

Table 1 highlights each of the physical, cognitive, and motor assessments performed in the study. Each of the indicated outcomes will be assessed before and after the 12-week interventions. This pre/post repeated measures design will be conducted on all randomized sedentary, older adult participants aged 65 to 80 years.

**Table 1:** Physical, cognitive, and motor assessments performed in the study.

Systems and assessment		Description	Outcomes		Component function		Relevant cortices	
**Cardiovascular-motor**								
	Modified Balke VO_2_ test	Treadmill test where participants walk at a constant 3 mph and treadmill grade is increased by 2.5% every 2 minutes until 90% of estimated heart rate max ((220-age)*0.90) is achieved.	Estimated VO_2_ max, peak VO_2_		Cardiovascular fitness, gross movement economy		Whole brain (gray matter)	
**Cognitive-executive**								
	MoCA^a^	This brief instrument asks a series of questions to screen for cognitive impairment.	Scaled score (0-30). A score <26 indicates cognitive impairment and is a study disqualifier.		Visual/executive, naming, memory, attention, abstraction, orientation		Frontal, temporal, parietal	
	Digit span forward/backward	Participants are provided a sequence of digits and prompted to immediately repeat the sequence as heard (forward) or in reverse order (backward). If repeated correctly, the next trial is presented with a longer sequence. The task ends with 3 incorrect response attempts at a given digit span.	Longest digit span repeated correctly		Working memory		Frontal, temporal, parietal	
	D-KEFS^b^: verbal fluency	Letter fluency: participants recall words beginning with a specified letter as quickly as possible. Category fluency: participants recall words belonging to the designated semantic category.	Number of words within 60 seconds		Letter fluency, semantic fluency		Frontal (IFG^c^)	
	D-KEFS: Color word interference (Stroop)	Inhibition: participants are presented with the words “red,” ”green,“ and ”blue“ printed incongruently in red, green, or blue font. They are instructed to state the font color as quickly as possible while minimizing mistakes. Switching: the same words with incongruent font colors are presented to participants; however, some words will be presented with a box outlining them. Participants are instructed to state the font color in nonoutlined text conditions and instead read each word aloud (as opposed to font color) when the word is outlined by a box.	Total time + (total time/100) x number of uncorrected errors		Response Inhibition, response switching		Frontal, temporal, parietal	
**Cognitive-motor**								
	D-KEFS: trail-making test	Participants are timed as they connect numbers and letters in ordered sequences	Time to completion (seconds)		Cognitive flexibility, visual sequence tracking		Visual, primary motor, frontal	
	Computerized N-back	Participants are provided a target letter before this task. They are then presented with a series of individual letters on a computer screen. They are instructed to denote, via keyboard response (1: “No”; 2: “Yes”), whether a presented letter corresponds with the target letter. The 0-back condition requires a response based on the letter immediately presented to them, and the 2-back condition requires a response based on the letter presented 2 steps behind the immediate letter presented.	Response accuracy (% correct), reaction Time (ms)		Working memory, processing speed, manual dexterity		Visual, primary motor, frontal	

^a^MoCA: Montreal Cognitive Assessment.

^b^D-KEFS: Delis-Kaplan Executive Function System.

^c^IFG: inferior frontal gyrus.

#### Day 2: MRI Environment

##### MRI Acquisition

MRI data will be collected on a 3T Siemens Prisma fit (Erlangen). This will consist of an anatomical T1-weighted MP-RAGE (magnetization-prepared rapid gradient-echo) scan (echo time = 3 ms, repetition time = 2500 ms, flip angle = 7^o^) and 7 fMRI scans for each participant: 1 fMRI scan collected during dual-echo pCASL (pseudocontinuous arterial spin labeling) (TE1/TE2 = 10/25 ms, TR = 4000 ms, 1 fMRI scan collected during CVR (TE = 21 ms, TR = 1000 ms, voxel size = 3mm x 3mm x 3mm, flip angle = 50^o^, number of volumes = 420), 3 runs of a language based task (TE = 25 ms, TR = 5800 ms, voxel size = 3 mm x 3 mm x 3 mm, flip angle = 70^o^, number of volumes = 71), and 2 runs of a motor based task (TE = 25 ms, TR = 2000 ms, voxel size = 3 mm x 3 mm x 3 mm, flip angle = 70^o^, number of volumes = 154).

Preprocessing

The preprocessing of anatomical T1w and fMRI data will be conducted using fMRI Prep 22.0.22 (RRID: SCR_016216), which is based on Nipype 1.8.5 (RRID: SCR_002502). The pipeline included brain extraction, tissue segmentation, and spatial normalization to the Montreal Neurological Institute (MNI) template space for the T1w. Regarding the fMRI data, preprocessing involved an estimation of the BOLD (blood oxygen level dependent) image and head-motion, slice timing correction, registration to the T1w image, and resampling to MNI space. Multimedia Appendix 1 contains the full preprocessing pipeline details.

##### Semantic Fluency

**Figure 1:**
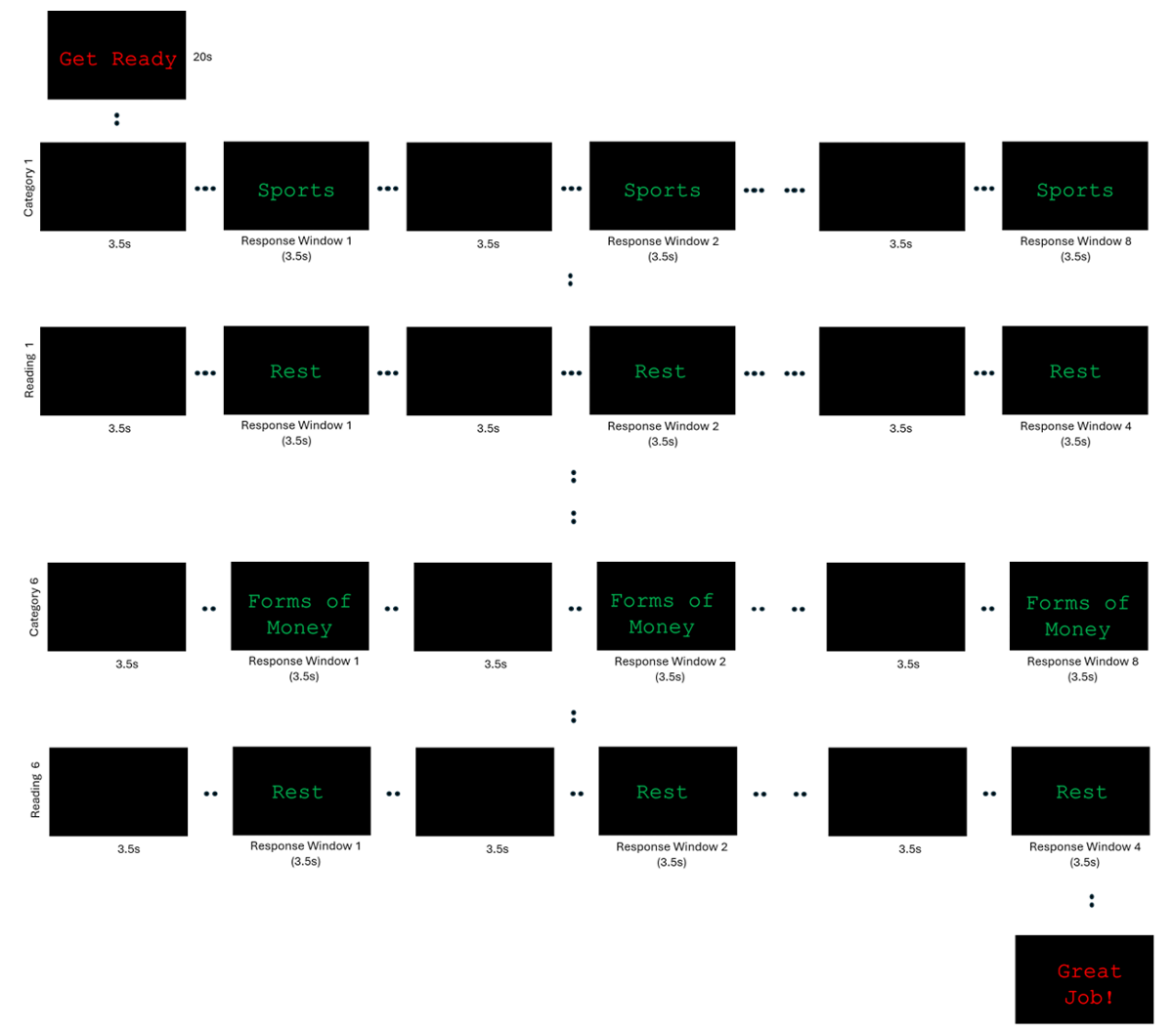
Visual representation of a single block of the semantic fluency task performed during task-dependent functional magnetic resonance imaging (fMRI). Each participant will perform the sematic fluency fMRI before and after the 12-week interventions. This pre/post repeated measures design will be conducted on all randomized sedentary, older adult participants (65-80 years).

During the MRI session, participants will perform 3 blocks of a verbal fluency task programmed using via E-Prime software (version 3.0, Psychology Software Tools) [13] (Figure 1). At the start of each block, the phrase “please wait” is projected to participants via a 20-inch HP computer display for 20 seconds. The task is initiated when this phrase is replaced by a word corresponding to a specific semantic category. This category disappears and reappears 8 separate times, and participants are instructed to verbalize a single response corresponding to this category each time it reappears on the screen. Participants are also instructed to avoid repeating any responses from the same category. A single task block consists of 6 separate categories (requiring 8 individuated/nonrepeating responses per category). Interleaved between each semantic category is a “reading” condition. Here, participants are presented with the word “rest” 4 separate times and instructed to verbally respond with the word “rest” each time it appears. During these scans, the verbal responses will be manually recorded by an investigator and subsequently scored based on the total number of correct (and nonrepeating) responses divided by the number of response windows provided. The block score will be calculated as the number of correct responses/48 response windows × 100, and the session score as the number of correct responses/144 response windows × 100.

##### Motor Tapping

**Figure 2:**
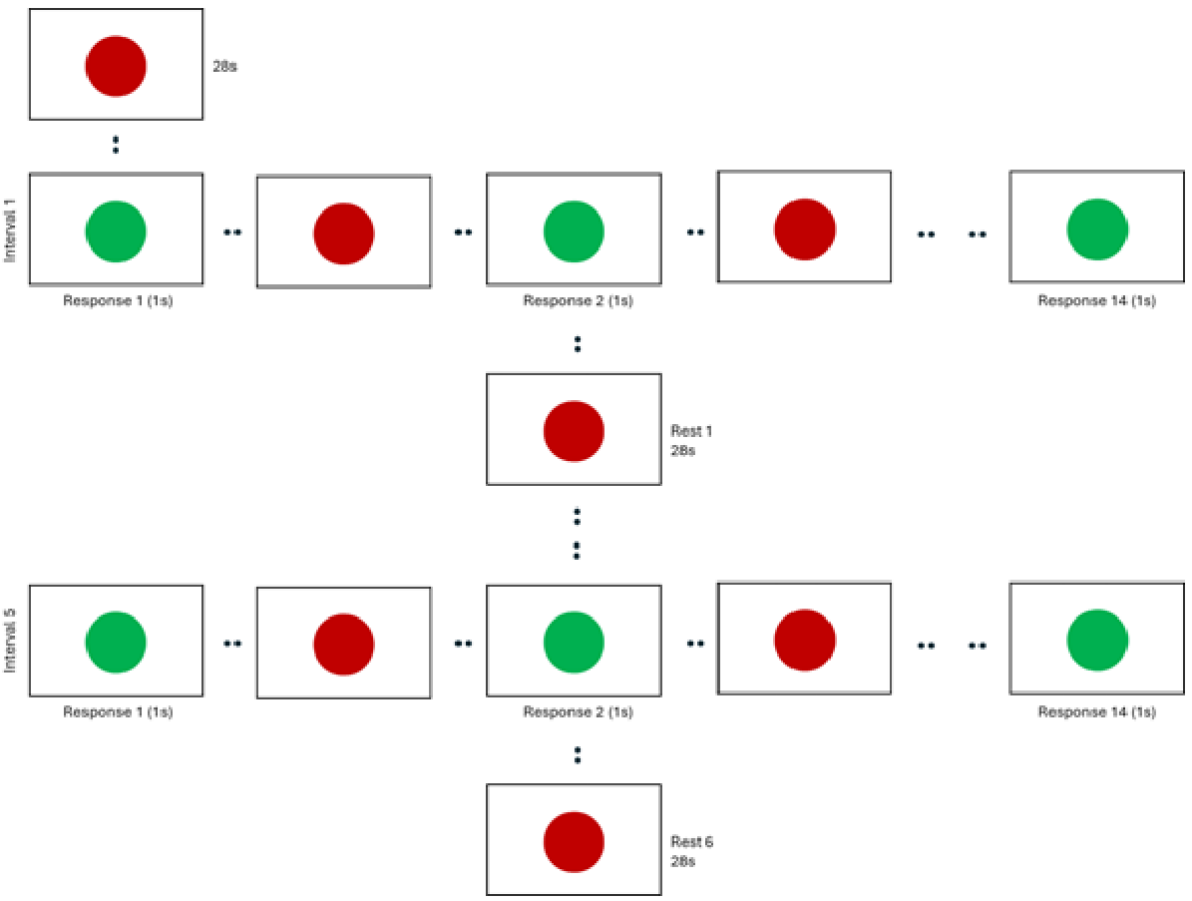
Visual representation of a single block of the motor tapping task performed during the functional magnetic resonance imaging (fMRI). Each participant will perform the sematic fluency fMRI before and after the 12-week interventions. This pre/post repeated measures design will be conducted on all randomized sedentary, older adult participants (65-80 years).

Participants will also perform 2 blocks of a motor tapping task programmed using PsychoPy [14] and using an MR-compatible 4-button box (MagConcept response unit) (Figure 2). Briefly, individuals are presented with a red dot in the center of the projected screen for 15 seconds. The task will commence when this dot turns green and subsequently alternates between red and green at a frequency of 1 Hz. Participants are instructed to press the button oriented to their index finger at the pace of the flashing green dot. Each block consists of five 28-second tapping intervals separated by six 28-second rest intervals. During these rest intervals, a solid red dot is projected, and participants are instructed to do nothing until they are presented with the next sequence of green dots.

The arterial spin labeling (ASL) sequence acquisition first magnetically labels blood water at the region of interest by applying a 180-degree radiofrequency inversion pulse. The labeled water exchanges with tissue water, which alters the tissue’s magnetization and the image intensity, creating a ”tag“ image. This process is repeated without labeling blood to create a ”control“ image. Subtracting the control and tag images produces perfusion imaging that reflects the amount of arterial blood delivered to voxels within a given region of interest. This difference signal is directly proportional to cerebral blood flow and can be mapped on a voxel-wise basis to obtain regional blood flow information. To convert the difference signal (=control-label) into physiological units, a single-compartment model is utilized to obtain units of mL/100g/min. The ASL scan lasts approximately 7 minutes.

The aforementioned sequence parameters to be used for ASL acquisition are standard for assessing blood flow maps of the whole brain, including our regions of interest (inferior frontal and the motor cortices). The Harvard-Oxford cortical atlas developed in the MNI 152 atlas space will be the starting point for the frontal region of interest. The resulting ASL with corresponding T1-weighted images will be registered into the MNI 152 atlas space using nonlinear algorithms from the Functional Magnetic Resonance Imaging of the Brain Software Library (FSL). The images will be imported into Analysis of Functional NeuroImages (AFNI) software (Scientific and Statistical Computing Core) for analysis.

CVR will be assessed using a block-design hypercapnic response paradigm where participants alternate between breathing room air and a 5% carbon dioxide (CO_2_) gas mixture during fMRI (see the detailed description in [15]). Participants will be fitted with a nose clip and mouthpiece attached to a 2-way breathing valve that affords inhalation of normal room air or a CO_2_ mixture filled in a 100 L Douglas bag. Additional physiological parameters, including end-tidal (Et) CO_2_ and breathing rate, will be continuously monitored and recorded using a Philips NM3 capnograph. At the onset of this scan, participants will inhale normal room air for 50 seconds. A research staff member will then manually switch the valve to CO_2_ air for 50 seconds and subsequently alternate between breathing conditions until three 50-second blocks of CO_2_ breathing and four 50-second blocks of room air breathing have been collected (total scan duration ~7 minutes).

Provided that Et-CO_2_ is essentially the input function to the brain vasculature, it is critical to measure this trace as it is a quantitative metric for the degree of stimulation the blood vessels receive. As such, the primary outcome from this measure is BOLD changes per mmHg of inhaled CO_2_ in our regions of interest.

### Ethical Considerations

All participants will provide informed consent before any testing commences. All study protocols will follow the Declaration of Helsinki and have been approved by the Emory University Internal Review Board and Atlanta Veteran Affairs (VA) Research and Development Office (RX002825-01). All interventions and assessments will take place at Emory University and Atlanta VA. Participants will be compensated US $150 for completing all pre/post assessments and the intervention. To ensure that all personal information collected during this study is kept strictly confidential, identifiable data will be stored securely and accessible only to authorized personnel involved in the research. Any published results will be presented in aggregate form, ensuring that individual participants cannot be identified. Participants' identities and sensitive information will be protected throughout and beyond the duration of this study, in accordance with ethical guidelines and legal requirements.

### Data Analyses

For data analyses, we aim to determine whether the 2 groups (spin and control) have different responses to cerebral perfusion in the regions of interest following the intervention. Our a priori hypothesis is that individuals in the aerobic group will show increased perfusion in both the right and left inferior frontal gyri and motor cortices when compared to those in the control group. The primary outcome is cerebral blood flow in units of mL/100 g/min within each region of interest. We will test each mL/100 g/min region of interest value jointly using a mixed effects linear model to determine whether the mean change of the groups is significantly different across the time points (pre and post). The fixed effect will be group (spin vs control), and the random effect will be time (pre vs post). We expect that modeling the outcomes jointly will allow for some gains in power. This model will enable us to estimate the overall effects of time and the interventions, and most importantly, how the effects of the interventions differ with time. Additional analyses will use multiple regressions to describe the effect of participant demographics (gender, race, age, and hypertensive status) or by preintervention cognitive status (MoCA). A false discovery rate (FDR) of q=0.05 will be calculated and used to correct for multiple tests, and effect sizes will be computed.

Next, we aim to determine the impact of an aerobic intervention on CVR. Our a priori hypothesis is that individuals in the aerobic group will show increased CVR in both the right and left inferior frontal gyri and motor cortices when compared to those in the control condition. Similar to our analyses for perfusion, we will test each region of interest BOLD change value jointly using a mixed effects linear model to determine whether the mean change of the groups is significantly different across the time points (pre and post). The fixed effect will be group (spin vs control) and the random effect will be time (pre vs post). Additional analyses will use multiple regressions to describe the effect of participant demographics (gender, race, age, and hypertensive status) or by preintervention cognitive function (MoCA). An FDR of q=0.05 will be calculated and used to correct for multiple tests, and effect sizes will be computed.

After the aforementioned preprocessing steps are performed on ASL and CVR, the images will be scaled to accurately determine the relative percent signal change in BOLD induced by the task. Finally, task-induced BOLD changes (ΔBOLD) from pre- to postaerobic intervention and corresponding hemodynamic response functions will be quantified using the deconvolution technique.

Since the goal of this aim is to quantify the impact of baseline physiologic measures (ie, resting blood flow and CVR on task-induced BOLD changes), we plan to accomplish this task by modeling the contributions of CBF and CVR to ΔBOLD responses. The following equations describe our simple modeling approach using linear regression:



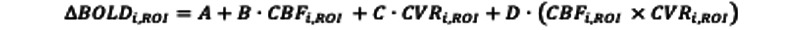



Where _i_=pre or post session, *ROI* refers to the regions of interest, and coefficients *A* is the modeled intercept, *B* and *C* are modeled slopes for perfusion and CVR contributions, and *D* is the modeled slope for the interaction between perfusion and CVR respectively.

We will compare the slopes obtained for pre- and postexercise periods to explore how each of the baseline measures (ie, CBF and CVR) changes (1) in response to exercise and (2) within each condition (pre or post). We also aim to disentangle the perfusion and vascular influences on task-induced BOLD changes.

Additionally, modeling for the interaction will account for the coupling effect, which can provide insights into exercise-induced perfusion-CVR coupled effects. We plan to carry out the processing and modeling at the ROI level as it renders more detection power and is computationally less intensive. From a statistics standpoint, we will compute the F score to test the significance of the overall fitting of the model, and we will also obtain the *t* test score for each predictor (perfusion and CVR) to test the significance of their association with changes in the measured variable (ΔBOLD).

The average area under the curve (AUC) will be taken from the right and left inferior frontal gyri ROIs during semantic fluency for both pre- and postsessions. The AUC will be averaged from the right and left inferior frontal gyri and treated as a single ROI for this analysis. The base analyses for this aim will be 2 analyses of variance (ANOVAs), 2 groups X 2 times (before vs after aerobic exercise).

The analysis plan for behavioral performance will include the following dependent variables: VO_2_, in-scanner verbal fluency performance, the motor task, as described previously, and our cognitive battery. Paired *t* tests will be performed to test for a pre-to-post effect. Spearman correlations (*r*) will be used to correlate behavioral performance to primary outcomes, and a mixed regression will be used to assess the influence of session (pre and post) on outcomes.

### Potential Outcomes

Decreased vascular health due to aging, particularly sedentary aging, fosters both cognitive and motor dysfunction [16], which has implications for neurodegenerative disease. Task-based fMRI and the resultant BOLD map are dominant methods of neurocognitive investigation, particularly related to documenting the beneficial changes demonstrated with rehabilitation. At the forefront of much of this research is the use of task-based fMRI BOLD to quantify beneficial changes in cortical function following aerobic exercise [11,17,18]. While transformative, the true impact of this research is limited in scope until we can define the influence of cerebrovascular function on the well-documented beneficial change in BOLD response. Because the BOLD signal reflects the health and function of the cerebrovasculature [19,20], we believe the changes brought on by exercise are at least partially mediated by improved perfusion and CVR. No single imaging technique can resolve the complexities of imaging neural plasticity in brain systems; therefore, we propose a solution of combining imaging techniques and modeling the impact of perfusion and CVR on the BOLD response. We will model the hypothesized change in perfusion and CVR to the BOLD response following the intervention to quantify the percentage of variance both perfusion and CVR account for in the task-induced BOLD signal. This will allow us to understand the degree to which change in perfusion and CVR impacts the well-documented beneficial change in task-induced BOLD following exercise.

## Results

This study was funded on December 3, 2018, by the Department of Veteran Affairs (grant RX004563-01A1). Final data was obtained by mid-2024, and the preliminary results of this study are expected to be published by early 2025. We expect the research to offer a clearer understanding of how aerobic exercise improves health and cerebrovascular function in older adults. We believe this will guide exercise prescriptions for older adults at risk of neurodegenerative diseases and provide stronger justification for large-scale implementation and expanded access to this low-cost intervention

## Discussion

### Aim and Previous Findings

This study aimed to explore the impact of aerobic exercise on cerebrovascular health in older adults. We know that older adults who are physically active have improved peripheral vascular health, but the impact of an exercise intervention on cerebrovascular health is less known. Therefore, we will fill this gap by examining changes in basal cerebral perfusion and CVR in older adults following a proven exercise intervention. If the hypotheses of improved perfusion and CVR are supported, it would inform intervention strategies and have implications for age-related disease brought on by cerebrovascular dysfunction.

The brain uses ~20% of available oxygen for normal function, making tight blood flow regulation and oxygen delivery critical for survival [21]. This high demand, coupled with a lack of energy stores within the cortex, necessitates that cerebral blood flow be constantly and consistently maintained. In a normal physiological state, total blood flow to the brain is remarkably constant; however, with advancing age, basal cerebral blood flow declines by roughly 4 mL min per year [22]. It is presumed reactive oxygen species in the cerebral vasculature decreases nitric oxide (NO) bioavailability leading to the gradual decline in endothelial function and subsequently perforating arteries, arterioles, and capillaries [18]. Ultimately, extracellular matrix components lose elasticity, causing decreased vessel function and decreased blood flow [23].

Morphological studies have demonstrated age-related degradation throughout the intracranial vessels. However, these studies have demonstrated that age-related decreases in perfusion are notably amplified in the frontal cortex [15]. It has been highlighted that age-related decline in cerebral perfusion is not great enough to cause major ischemic injury but leads to hypoperfusion that prevents sufficient nutrients from reaching highly metabolically demanding areas (eg, frontal cortex) [24]. Of note, the decreased blood supply and inadequate delivery of nutrients is noted as a key physiologic process in age-related cognitive decline and specifically cognitive-executive processes partially mediated by the frontal cortex [25].

### Impact of Exercise on Cerebral Blood Flow

Studies have focused on the beneficial effects of exercise on age-related changes in blood flow in the periphery, but less is known about aging and exercise on cerebral perfusion. In the periphery, exercise with an aerobic component is associated with improved perfusion in older adults compared to their sedentary peers [18]. Specific to the cerebral cortex, a cross-sectional study showed blood flow velocity in the middle cerebral artery, probed using Doppler, was significantly higher in adults with high levels of cardiovascular fitness [26]. Of note, the decrease in basal cerebral blood flow is tapered in people who report higher levels of physical activity [27]. Although these data give us an indication that aerobic exercise can enhance cerebral perfusion, it is currently unknown whether a proven aerobic intervention in previously sedentary older adults can benefit basal cerebral perfusion.

### Decreased Cerebrovascular Reactivity in Aging

In addition to the static parameter of blood flow, the dynamic parameter of CVR has a critical role in cerebrovascular health and function*.* The cerebral vasculature must maintain blood flow within precise limits during rest, activity, disease, or injury. In healthy individuals, the cerebral blood flow is tightly regulated to meet the metabolic demands of the brain via vasoconstriction or vasodilation. Thus, the ability of cerebrovasculature to dilate in the face of increased demand is vital for cerebral function. However, the vasoproperties and vascular response to demand have repeatedly been demonstrated to degrade with age and neuropathology [6,18]. Most often, to quantify the efficiency of cerebrovascular response, the degree of vasodilation in response to a vasoactive agent (CO_2_) is measured. Decreased responsiveness to CO_2_ is a proven marker of CVR dysfunction and has been demonstrated to be sensitive to changes brought on by aging and neural disease [15,27].

While the mechanisms are difficult to directly probe in human cerebrovasculature, extensive work in the periphery demonstrates that age-related arterial stiffening and endothelial dysfunction play critical roles in the development of CVR dysfunction [18]. Impairment of the oxidative stress response and dysfunctional inflammatory pathways feed a cascade of age-related CVR malfunction. Dysfunction in the stress response and inflammatory pathways causes increased reactive oxygen species and reduced NO. Together, the increase in reactive oxygen species and decrease NO suppresses the response of the immune cells, causing systemic vascular endothelial dysfunction [28-30]. Behaviorally, decreased CVR has been observed in cognitive decline demonstrated in aging as well as dementia [31].

### Impact of Exercise on Cerebral Blood Flow

Aerobic exercise has consistently been associated with enhanced flow-mediated dilatation in the brachial artery of older adults [32]. In the cortex, vasodilation of the cerebrovascular in response to a CO_2_ challenge is associated with maximal aerobic capacity in both young and old adults [5,6,33]. Thus, in both the periphery and the cortex, those with higher levels of cardiovascular fitness have improved CVR to stimulus. CVR was also shown to increase among stroke survivors after 6 months of aerobic training [26]. Our own pilot data, described in greater detail in the subsequent paragraphs, demonstrate a marked decline in CVR with age. However, the degree of CVR decay is lessened in older adults who have high levels of self-reported aerobic activity. Thus, the purpose of this study is a key next step toward quantifying CVR change following a proven aerobic intervention in previously sedentary older adults.

### Link Between Cerebral Perfusion, CVR, and Task-Based fMRI BOLD Response

Seminal studies have continually demonstrated that previously sedentary individuals who undergo exercise intervention show BOLD activity that coincides with improvements in an array of tasks in both motor and cognitive domains [7,11,34]. While the accessibility and ease of implementation have made BOLD a valuable tool, its interpretation is not straightforward [20]. BOLD is a convoluted neurovascular signal that arises due to the interplay between changes in blood flow and metabolism in response to inhibitory/excitatory stimuli (Figure 2). Task-induced BOLD change (ΔBOLD) is measured relative to the baseline BOLD signal, which means that baseline physiology (blood flow and perfusion; red box in Figure 2) affects the task-induced ΔBOLD (especially magnitude). We believe improvements in these baseline physiological factors are at least partially responsible for the well-documented, exercise-induced BOLD change. As noted, precise levels of blood flow depend on the health of the blood vessels that carry them. Blood carries important nutrients such as glucose and oxygen to support cellular energy demands; thus, the amount of baseline cerebral blood flow is an indirect measure of tissue baseline metabolic demand. It should be noted that perfusion is a proxy for baseline neural activity, as increased neural activity results in increased demand for blood flow [35]. Further, the blood vessels must dilate in response to an increased demand for blood flow. CVR is an index of baseline vascular health that is necessary to support efficient cerebral perfusion. Thus, to tease out the issue of vascular “plumbing” versus lower baseline neural activity, we need to quantify both perfusion and CVR. Taken together, for a better interpretation of task-induced ΔBOLD, it is important to understand the relationship between baseline cerebral physiology (perfusion and CVR) and task-induced ΔBOLD. To better understand the literature highlighting BOLD alterations following exercise, it is critical to quantify the impact of baseline physiologic measures, including perfusion and CVR on task-induced BOLD changes. We plan to accomplish this task by modeling the contributions of perfusion and CVR to ΔBOLD response both before and after the intervention.

### Limitations

We have considered the following potential weaknesses. First, the length of the intervention (12 weeks) is not sufficient to induce change in cerebrovascular function*.* Of note, we encourage all our research participants to maintain a lifelong commitment to physical activity and hope that our 12-week intervention is the beginning of behavioral change. We do believe, however, that the 12-week timeline is sufficient to significantly change both perfusion and reactivity based on (1) the significant change demonstrated in cardiovascular fitness and BOLD response in 12 weeks utilizing the proposed spin intervention, and (2) numerous studies demonstrating changes in perfusion and reactivity in the periphery with 12-week interventions. Further, it is known that acute bouts of moderate-intensity aerobic exercise reduce both central and peripheral femoral artery stiffness [36], while increasing endothelial function [37,38] has anti-inflammatory benefits.

An additional potential weakness is that older adults will not be compliant and adherent to the intervention. Compliance in our studies generally has been at or above 90% with retention consistently being above 85%; however, the long-term success of interventions will depend on accessibility and sustainability. Community exercise programs that increase accessibility are yielding positive results [39], but sustainability is still an issue. Dealing with these issues in later-stage clinical trials is in our long-term goals once we know the full extent of the intervention and it is consistent with our long-term goals.

### Conclusion

We believe this study will impact older adults and the state of science by defining and quantifying the positive impact of aerobic exercise on cerebrovascular health. Decreased vascular health because of aging, and particularly sedentary aging, lays the groundwork for both cognitive and motor dysfunction and is linked to neurodegenerative disease. Cross-sectional works and our preliminary data suggest that our aerobic exercise intervention will improve cerebrovascular health. If our hypotheses are supported, it would inform current intervention strategies and would add important new information on the implications of the cerebrovasculature on the BOLD signal. These findings would advance the current literature regarding the beneficial impact of exercise on brain health and function.

In terms of future direction, our lab has repeatedly shown that neurologic changes brought on by aging and disease may not be inevitable or immutable. We have new evidence that indicates increased levels of physical fitness through aerobic activity may mitigate losses in interhemispheric inhibition. We anticipate that cerebrovascular health in aging is intertwined with levels of interhemispheric inhibition, and both will improve throughout our exercise intervention. The next steps for our lab would be to further develop a continuum of research investigating the relationship between neurodegenerative disease, cerebrovascular reactivity, and physical activity, providing a cornerstone to build on our understanding of cerebrovascular disease processes and opportunities to prevent, intervene, or reverse.

## Appendix

Multimedia Appendix 1 Preprocessing pipeline details.

## Abbreviations

AD: Alzheimer disease
AFNI: Analysis of Functional Neuroimages
ANOVA: analyses of variance
AUC: area under the curve
BOLD: blood oxygen level dependent
CO_2_: carbon dioxide
CVR: cerebral vascular reactivity
Et: end tidal
FDR: false discovery rate
fMRI: functional magnetic resonance imaging
FSL: Functional Magnetic Resonance Imaging of the Brain Software Library
MNI: Montreal Neurological Institute
MoCA: Montreal Cognitive Assessment Score
MP-RAGE: magnetization-prepared rapid gradient-echo
MRI: magnetic resonance imaging
NO: nitric oxide
pCASL: pseudocontinuous arterial spin labeling
VA: Veteran Affairs

## Data Availability

Data will be shared in accordance with the Department of Veteran Affairs Rehabilitation Research policy.

## References

[1] Alzheimer's Association (2013). 2013 Alzheimer's disease facts and figures. Alzheimers Dement.

[2] Attems J, Jellinger KA (2014). The overlap between vascular disease and Alzheimer's disease--lessons from pathology. BMC Med.

[3] Toledo J, Arnold S, Raible K, Brettschneider J, Xie SX, Grossman M, Monsell SE, Kukull WA, Trojanowski JQ (2013). Contribution of cerebrovascular disease in autopsy confirmed neurodegenerative disease cases in the National Alzheimer's Coordinating Centre. Brain.

[4] Sperling R, Mormino E, Johnson K (2014). The evolution of preclinical Alzheimer's disease: implications for prevention trials. Neuron.

[5] Barnes DE, Yaffe K (2011). The projected effect of risk factor reduction on Alzheimer's disease prevalence. Lancet Neurol.

[6] Barnes JN (2015). Exercise, cognitive function, and aging. Adv Physiol Educ.

[7] Colcombe S, Kramer AF (2003). Fitness effects on the cognitive function of older adults: a meta-analytic study. Psychol Sci.

[8] Kramer AF, Hahn S, Cohen NJ, Banich MT, McAuley E, Harrison CR, Chason J, Vakil E, Bardell L, Boileau RA, Colcombe A (1999). Ageing, fitness and neurocognitive function. Nature.

[9] Voss MW, Erickson KI, Prakash RS, Chaddock L, Kim JS, Alves H, Szabo A, Phillips SM, Wójcicki TR, Mailey EL, Olson EA, Gothe N, Vieira-Potter VJ, Martin SA, Pence BD, Cook MD, Woods JA, McAuley E, Kramer AF (2013). Neurobiological markers of exercise-related brain plasticity in older adults. Brain Behav Immun.

[10] McGregor KM, Crosson B, Krishnamurthy LC, Krishnamurthy V, Hortman K, Gopinath K, Mammino KM, Omar J, Nocera JR (2018). Effects of a 12-week aerobic spin intervention on resting state networks in previously sedentary older adults. Front Psychol.

[11] Nocera J, Crosson B, Mammino K, McGregor KM (2017). Changes in cortical activation patterns in language areas following an aerobic exercise intervention in older adults. Neural Plast.

[12] Nocera JR, Mammino K, Kommula Y, Wharton W, Crosson B, McGregor KM (2020). Effects of combined aerobic exercise and cognitive training on verbal fluency in older adults. Gerontol Geriatr Med.

[13] Smith RE, Hunt RR (1998). Presentation modality affects false memory. Psychon Bull Rev.

[14] Peirce J, Gray JR, Simpson S, MacAskill M, Höchenberger R, Sogo H, Kastman E, Lindeløv JK (2019). PsychoPy2: Experiments in behavior made easy. Behav Res Methods.

[15] Lu H, Liu P, Yezhuvath U, Cheng Y, Marshall O, Ge Y (2014). MRI mapping of cerebrovascular reactivity via gas inhalation challenges. J Vis Exp.

[16] McGregor KM, Zlatar Z, Kleim E, Sudhyadhom A, Bauer A, Phan S, Seeds L, Ford A, Manini TM, White KD, Kleim J, Crosson B (2011). Physical activity and neural correlates of aging: a combined TMS/fMRI study. Behav Brain Res.

[17] Colcombe SJ, Kramer AF, Erickson KI, Scalf P, McAuley E, Cohen NJ, Webb A, Jerome GJ, Marquez DX, Elavsky S (2004). Cardiovascular fitness, cortical plasticity, and aging. Proc Natl Acad Sci USA.

[18] Santos-Parker JR, LaRocca TJ, Seals DR (2014). Aerobic exercise and other healthy lifestyle factors that influence vascular aging. Adv Physiol Educ.

[19] Erickson KI, Gildengers AG, Butters MA (2022). Physical activity and brain plasticity in late adulthood. Dialogues Clin Neurosci.

[20] Halani S, Kwinta JB, Golestani AM, Khatamian YB, Chen JJ (2015). Comparing cerebrovascular reactivity measured using BOLD and cerebral blood flow MRI: The effect of basal vascular tension on vasodilatory and vasoconstrictive reactivity. Neuroimage.

[21] Attwell D, Buchan AM, Charpak S, Lauritzen M, Macvicar BA, Newman EA (2010). Glial and neuronal control of brain blood flow. Nature.

[22] Stoquart-ElSankari S, Balédent Olivier, Gondry-Jouet C, Makki M, Godefroy O, Meyer M (2007). Aging effects on cerebral blood and cerebrospinal fluid flows. J Cereb Blood Flow Metab.

[23] Ungvari Z, Kaley G, de Cabo R, Sonntag WE, Csiszar A (2010). Mechanisms of vascular aging: new perspectives. J Gerontol A Biol Sci Med Sci.

[24] Iadecola C (2004). Neurovascular regulation in the normal brain and in Alzheimer's disease. Nat Rev Neurosci.

[25] Davenport M, Hogan DB, Eskes GA, Longman RS, Poulin MJ (2012). Cerebrovascular reserve: the link between fitness and cognitive function?. Exerc Sport Sci Rev.

[26] Akazawa N, Choi Y, Miyaki A, Tanabe Y, Sugawara J, Ajisaka R, Maeda S (2012). Curcumin ingestion and exercise training improve vascular endothelial function in postmenopausal women. Nutr Res.

[27] Thomas BP, Yezhuvath US, Tseng BY, Liu P, Levine BD, Zhang R, Lu H (2013). Life-long aerobic exercise preserved baseline cerebral blood flow but reduced vascular reactivity to CO2. J Magn Reson Imaging.

[28] Lakatta EG, Levy D (2003). Arterial and cardiac aging: major shareholders in cardiovascular disease enterprises: Part II: the aging heart in health: links to heart disease. Circulation.

[29] LaRocca TJ, Hearon CM, Henson GD, Seals DR (2014). Mitochondrial quality control and age-associated arterial stiffening. Exp Gerontol.

[30] LaRocca TJ, Henson GD, Thorburn A, Sindler AL, Pierce GL, Seals DR (2012). Translational evidence that impaired autophagy contributes to arterial ageing. J Physiol.

[31] den Abeelen ASSM, Lagro J, van Beek AHEA, Claassen J (2014). Impaired cerebral autoregulation and vasomotor reactivity in sporadic Alzheimer's disease. Curr Alzheimer Res.

[32] Mora S, Cook N, Buring JE, Ridker PM, Lee I (2007). Physical activity and reduced risk of cardiovascular events. Circulation.

[33] Bailey DM, Marley CJ, Brugniaux JV, Hodson D, New KJ, Ogoh S, Ainslie PN (2013). Elevated aerobic fitness sustained throughout the adult lifespan is associated with improved cerebral hemodynamics. Stroke.

[34] Erickson KI, Gildengers AG, Butters MA (2022). Physical activity and brain plasticity in late adulthood. Dialogues Clin Neurosci.

[35] Buxton RB (2010). Interpreting oxygenation-based neuroimaging signals: the importance and the challenge of understanding brain oxygen metabolism. Front Neuroenergetics.

[36] Kingwell BA, Berry KL, Cameron JD, Jennings GL, Dart AM (1997). Arterial compliance increases after moderate-intensity cycling. Am J Physiol Heart Circ Physiol.

[37] Rooks CR, McCully KK, Dishman RK (2011). Acute exercise improves endothelial function despite increasing vascular resistance during stress in smokers and nonsmokers. Psychophysiology.

[38] Whyte JJ, Laughlin MH (2010). The effects of acute and chronic exercise on the vasculature. Acta Physiol (Oxf).

[39] Desveaux L, Beauchamp M, Goldstein R, Brooks D (2014). Community-based exercise programs as a strategy to optimize function in chronic disease: a systematic review. Med Care.

