# Supplementary material for: Graded Intensity Aerobic Exercise to Improve Cerebrovascular Function and Performance in Older Veterans: Protocol for a Randomized Controlled Trial
Source: JMIR Res Protoc. 2024 Sep 26;13:e58316. doi: 10.2196/58316 (PMC11467598; doi:10.2196/58316)
Supplement: Multimedia Appendix 1 [file resprot_v13i1e58316_app1.docx]

| **System(s)** | **Assessment** | **Description** | **Outcomes** | **Component Function** | **Relevant Cortices** |
| --- | --- | --- | --- | --- | --- |
| Cardiovascular, Motor | Modified Balke VO2 test | Treadmill test where participants walk at a constant 3mph, and treadmill grade is increased by 2.5% every 2 minutes until 90% of estimated heart rate max ((220-age)*0.90) is achieved. | Estimated VO2 max, Peak VO2 | Cardiovascular fitness, gross movement economy | Whole brain (gray matter) |
| Cognitive-Executive | Montreal Cognitive Assessment (MoCA) | This brief instrument asks a series of questions to screen for cognitive impairment. | Scaled score (0-30). < 26 indicates cognitive impairment and is a study disqualifier. | Visual/Executive, naming, memory, attention, abstraction, orientation | Frontal, Temporal, Parietal |
|  | Digit span forward/backward | Participants are provided a sequence of digits and prompted to immediately repeat the sequence as heard (forward), or in reverse order (backwards). If repeated correctly, the next trial is presented with a longer sequence. The task ends with 3 incorrect response attempts at a given digit span. | Longest digit span repeated correctly | Working memory | Frontal, Temporal, Parietal |
|  | D-KEFS: Verbal fluency | Letter fluency- participants recall words beginning with a specified letter as quickly as possible.  Category fluency- recall words belonging to designated semantic category. | # of words within 60s | Letter fluency, semantic fluency | Frontal (Inferior Frontal Gyrus (IFG) |
|  | D-KEFS: Color word interference (Stroop) | Inhibition- Participants are presented with the words "red", "green", "blue" printed incongruently in red, green, or blue font. They are instructed to state the font color as quickly as possible while minimizing mistakes.  Switching- The same words with incongruent font color are presented to participants, however, some words will be presented with a box outlining them. Participants are instructed to state the font color in non-outlined text conditions, and instead read each word aloud (as opposed to font color) when the word is outlined by a box. | Total time + (total time/100) x # uncorrected errors | Response Inhibition, response switching | Frontal, Temporal, Parietal |
| Cognitive, Motor | D-KEFS: Trail making test | Participants are timed as they connect numbers and letters in ordered sequences | Time to completion (seconds) | Cognitive flexibility, visual sequence tracking | Visual, Primary Motor, Frontal |
|  | Computerized N-back | Participants are provided a target letter before this task. They are then presented with a series of individual letters on a computer screen. They are instructed to denote, via keyboard response (1-“No”, 2-“Yes”), whether a presented letter corresponds with the target letter. 0-back condition requires response based on the letter immediately presented to them, and 2-back condition requires response based on the letter presented 2 steps behind the immediate letter presented. | Response accuracy (% correct), Reaction Time (ms) | Working memory, processing speed, manual dexterity | Visual, Primary Motor, Frontal |


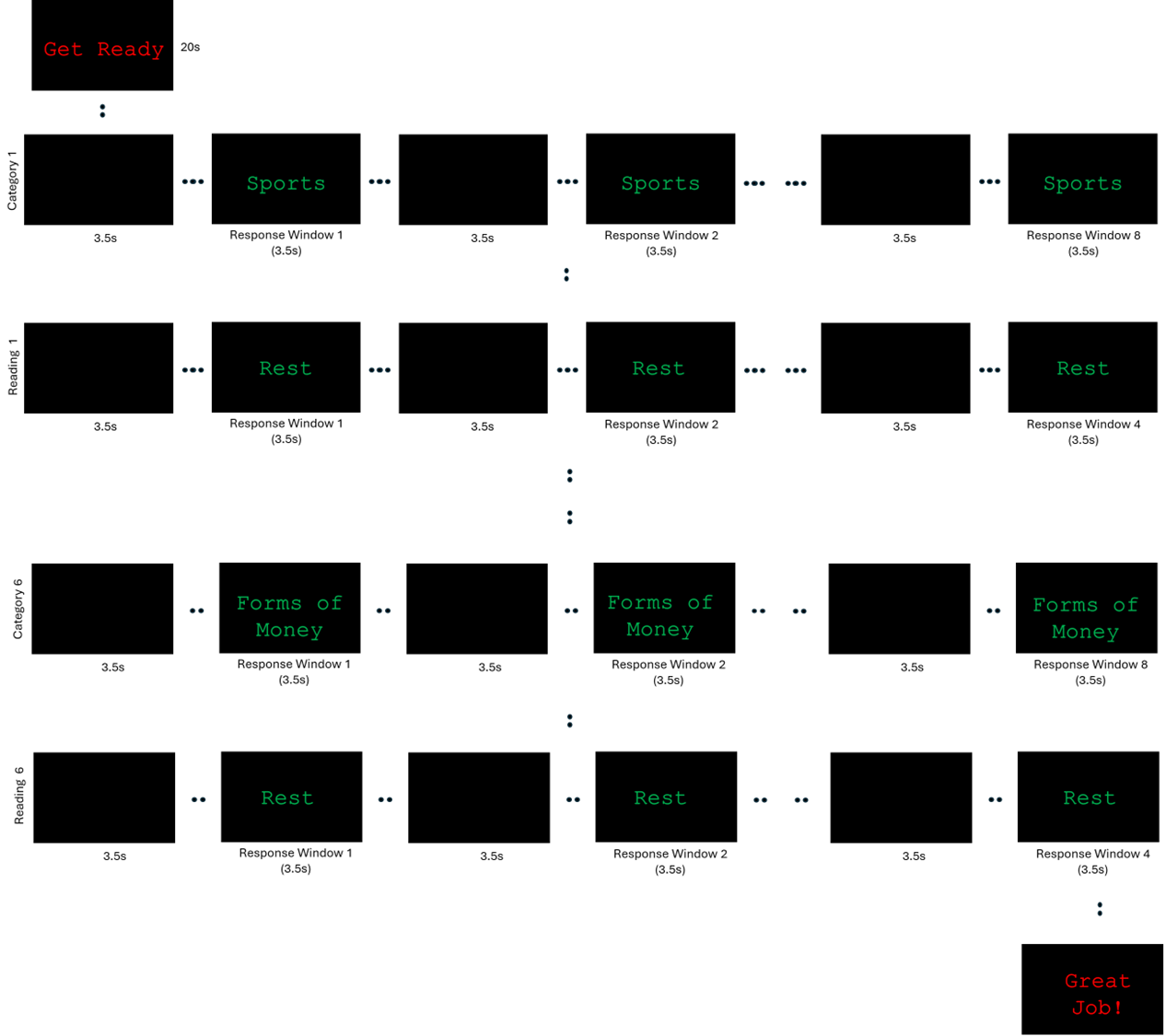


Figure 1. Visual representation of a single block of the semantic fluency task performed during fMRI.


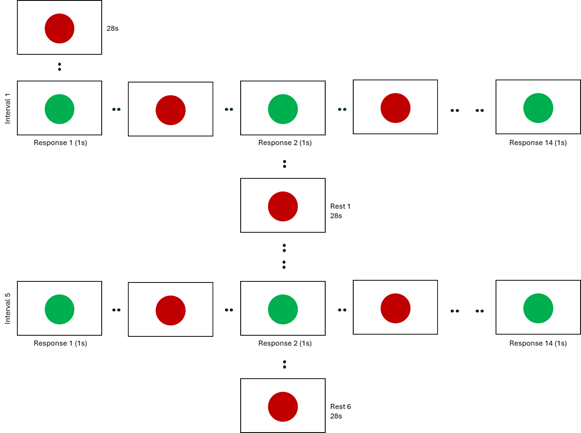


Figure 2. Visual representation of a single block of the motor tapping task performed during fMRI.
